# Effect of Maternal Obstructive Sleep Apnea-Hypopnea on 24-Hour Blood Pressure, Nocturnal Blood Pressure Dipping and Arterial Stiffness in Hypertensive Disorders of Pregnancy

**DOI:** 10.3389/fphys.2021.747106

**Published:** 2021-10-18

**Authors:** Pattaraporn Panyarath, Noa Goldscher, Sushmita Pamidi, Stella S. Daskalopoulou, Robert Gagnon, Natalie Dayan, Kathleen Raiche, Allen Olha, Andrea Benedetti, R. John Kimoff

**Affiliations:** ^1^Respiratory Division and Sleep Laboratory, McGill University Health Centre, Montreal, QC, Canada; ^2^Division of Respiratory and Respiratory Critical Care Medicine, Department of Internal Medicine, Faculty of Medicine, Prince of Songkla University, Songkhla, Thailand; ^3^Center for Outcomes Research, McGill University Health Centre, Montreal, QC, Canada; ^4^Division of Internal Medicine, McGill University Health Centre, Montreal, QC, Canada; ^5^Maternal-Fetal Medicine, Department of Obstetrics and Gynecology, McGill University Health Centre, Montreal, QC, Canada; ^6^Department of Epidemiology and Biostatistics, McGill University Health Centre, Montreal, QC, Canada

**Keywords:** obstructive sleep apnea-hypopnea, 24-h blood pressure, nocturnal blood pressure dipping, hypertensive disorders of pregnancy, arterial stiffness

## Abstract

**Rationale:** Maternal obstructive sleep apnea-hypopnea (OSAH) is associated with hypertensive disorders of pregnancy (HDP). Attenuation of the normal nocturnal blood pressure (BP) decline (non-dipping) is associated with adverse pregnancy outcomes. OSAH is associated with nocturnal non-dipping in the general population, but this has not been studied in pregnancy. We therefore analyzed baseline data from an ongoing RCT (NCT03309826) assessing the impact of OSAH treatment on HDP outcomes, to evaluate the relationship of OSAH to 24-h BP profile, in particular nocturnal BP dipping, and measures of arterial stiffness.

**Methods:** Women with a singleton pregnancy and HDP underwent level II polysomnography. Patients with OSAH (apnea-hypopnea index (AHI) ≥ 5 events/h) then underwent 24-h ambulatory BP monitoring and arterial stiffness measurements (applanation tonometry, SphygmoCor). Positive dipping was defined as nocturnal systolic blood pressure (SBP) dip ≥ 10%. The relationships between measures of OSAH severity, measures of BP and arterial stiffness were evaluated using linear regression analyses.

**Results:** We studied 51 HDP participants (36.5 ± 4.9 years, BMI 36.9 ± 8.6 kg/m^2^) with OSAH with mean AHI 27.7 ± 26.4 events/h at 25.0 ± 4.9 weeks’ gestation. We found no significant relationships between AHI or other OSA severity measures and mean 24-h BP values, although BP was generally well-controlled. Most women were SBP non-dippers (78.4%). AHI showed a significant inverse correlation with % SBP dipping following adjustment for age, BMI, parity, gestational age, and BP medications (β = −0.11, *p* = 0.02). Significant inverse correlations were also observed between AHI and DBP (β = −0.16, *p* = 0.01) and MAP (β = −0.13, *p* = 0.02) % dipping. Oxygen desaturation index and sleep time below SaO_2_ 90% were also inversely correlated with % dipping. Moreover, a significant positive correlation was observed between carotid-femoral pulse wave velocity (cfPWV) and REM AHI (β = 0.02, *p* = 0.04) in unadjusted but not adjusted analysis.

**Conclusion:** Blood pressure non-dipping was observed in a majority of women with HDP and OSAH. There were significant inverse relationships between OSAH severity measures and nocturnal % dipping. Increased arterial stiffness was associated with increasing severity of OSAH during REM sleep in unadjusted although not adjusted analysis. These findings suggest that OSAH may represent a therapeutic target to improve BP profile and vascular risk in HDP.

## Introduction

Hypertensive disorders of pregnancy (HDP) complicate up to 10% of pregnancies and are a leading cause of maternal and infant morbidity and mortality worldwide (Lo et al., 2013; Seely and Ecker, 2014; Folk, 2018; Shah and Gupta, 2019). HDP are classified by the American College of Obstetricians and Gynecologists (ACOG) into four categories: pre-eclampsia–eclampsia, chronic hypertension, chronic hypertension with superimposed pre-eclampsia, and gestational hypertension. Chronic hypertension is defined as hypertension that develops either pre-pregnancy or at less than 20 weeks’ gestation. Gestational hypertension is defined as blood pressure (BP) higher than 140/90 mmHg diagnosed after 20 weeks of gestation, and pre-eclampsia is characterized by new-onset hypertension with proteinuria or end organ damage symptoms (Roberts et al., 2013).

Nocturnal BP dipping is a reflection of the normal physiologic reduction in sympathetic nervous activity during sleep (O’Brien et al., 2013; Parati et al., 2014). Normally, night-time systolic and diastolic BP are 10–20% lower than daytime BP in non-pregnant women and throughout normal pregnancy (Gupta et al., 2011). However, many patients with HDP demonstrate an absence of nocturnal BP dipping (non-dippers) (Oney and Meyer-Sabellek, 1990; Ayala et al., 1997; Hermida et al., 2000; Gupta et al., 2011). In the general population, non-dipping predicts mortality and cardiovascular events (Lee et al., 2005; Fagard et al., 2009; Gavriilaki et al., 2020). In HDP, an association between BP non-dipping and altered hemodynamic function and fetal growth restriction (FGR) has been reported. Ilic et al. (2017) demonstrated that the prevalence of preterm delivery and FGR was significantly higher in non-dippers compared to dippers (66.7% vs. 16.7% for preterm delivery and 72.4% vs. 24.2% for FGR). Furthermore, the authors found that non-dipping in HDP patients was associated with impaired maternal cardiac function, including reduced left ventricle ejection fraction, velocity of longitudinal systolic function, and cardiac output (Ilic et al., 2017). Targeting factors associated with BP non-dipping could, therefore, potentially contribute to improved maternal and fetal outcomes in HDP.

Obstructive sleep apnea-hypopnea (OSAH) is characterized by repetitive upper airway obstruction during sleep resulting in intermittent hypoxia and sleep fragmentation (American Academy of Sleep Medicine Task Force, 1999). OSAH is associated with sympathetic activation, oxidative stress, systemic inflammation, and endothelial dysfunction, and in the general population is associated with hypertension and increased risk of congestive heart failure, and acute ischemic events (Peppard et al., 2000; Baguet et al., 2012; Phillips and O’Driscoll, 2013). OSAH in the general population is also associated with BP non-dipping (Seif et al., 2014; Torres et al., 2015; Genta-Pereira et al., 2018; Crinion et al., 2019; Cuspidi et al., 2019; Pio-Abreu et al., 2021). The reported prevalence of a non-dipping BP pattern in OSAH is 48–84% and a recent meta-analysis study found that OSAH increases the likelihood of BP non-dipping by 1.5-fold (Cuspidi et al., 2019). Crinion et al. (2019) reported a significant inverse relationship between OSAH severity and the magnitude of nocturnal BP dip (β = −0.29, *p* = 0.03). On the other hand, randomized, controlled trials have demonstrated that treatment of OSAH with continuous positive airway pressure (CPAP) lowers BP in non-pregnant people with hypertension (Bratton et al., 2015; Patil et al., 2019). Of note, BP non-dipping was identified as a good predictor of BP response to CPAP treatment in people with OSAH (Labarca et al., 2021).

Obstructive sleep apnea-hypopnea may develop or worsen over the course of pregnancy, reaching an estimated prevalence of 17–45% of women in the third trimester (Pamidi and Kimoff, 2018). There is increasing evidence that maternal OSAH is associated with a greater risk of HDP (Champagne et al., 2009; Ding et al., 2014; Pamidi et al., 2014; Bourjeily et al., 2017; Li et al., 2018; Liu et al., 2019; Querejeta Roca et al., 2020; Lu et al., 2021) and other adverse pregnancy outcomes, including gestational diabetes mellitus and small for gestational age infants (SGA) (Pamidi et al., 2016). Recent meta-analyses have reported adjusted odds ratios of 1.93–2.38 for the association between OSAH and gestational hypertension (Ding et al., 2014; Pamidi et al., 2014; Li et al., 2018; Liu et al., 2019; Lu et al., 2021). Similarly, OSAH is associated with increased risk for pre-eclampsia (adjusted odds ratios of 2.19–2.63) (Ding et al., 2014; Pamidi et al., 2014; Li et al., 2018; Liu et al., 2019; Lu et al., 2021). The findings from the general adult OSAH population suggest that maternal OSAH may also be associated with non-dipping in HDP, and thus potentially contribute to the links between maternal BP non-dipping and adverse HDP outcomes. However, this has not been directly studied.

Arterial stiffness is a summative indicator of vascular health and an established cardiovascular risk marker in the general population. Previous studies have demonstrated that OSAH in the general population is associated with increased arterial stiffness and that stiffness measures improve with CPAP treatment (Kohler et al., 2013; Vlachantoni et al., 2013; Wons and Kohler, 2015; Lin et al., 2016; Chalegre et al., 2020). Arterial stiffness is increased in HDP and a previous meta-analysis (Hausvater et al., 2012) found significantly higher values for carotid-femoral pulse wave velocity (cfPWV) and augmentation index (AIx) in pre-eclampsia compared with gestational hypertension. Work at our center has also recently shown that changes in arterial stiffness predict subsequent development of pre-eclampsia (Phan et al., 2021). The foregoing observations raise the possibility that maternal OSAH may also be associated with increased arterial stiffness in HDP. However, this has not been directly studied.

The primary aim of this study was therefore to evaluate the relationship of maternal OSAH to 24-h BP and in particular nocturnal BP dipping in women with HDP. We also aimed to evaluate the relationship of OSAH to measures of arterial stiffness in women with HDP with OSAH.

## Materials and Methods

### Study Design

This is a cross-sectional analysis of baseline data collected from an ongoing pilot randomized controlled trial (RCT) assessing the impact of OSAH treatment on HDP outcomes. The RCT is registered at clinicaltrials.gov (NCT03309826). The primary objective of this study was to evaluate the relationship of maternal OSAH to 24-h BP and in particular, nocturnal BP dipping in women with HDP. The secondary objective was to evaluate the relationship of OSAH to measures of arterial stiffness in women with HDP.

### Study Population and Protocol

We recruited women ≥18 years of age with a singleton pregnancy, ≥12 weeks’ gestation diagnosed with hypertension defined by daytime systolic BP ≥ 140 and/or diastolic BP ≥ 90 mmHg or current antihypertensive treatment from our obstetric clinics. Women were excluded if they had current severe pre-eclampsia/eclampsia requiring immediate delivery; chronic kidney disease (serum creatinine >100 mmol/L); another secondary cause of hypertension; active cardiac disease or stroke within the last 3 months; malignancy or other active psychiatric or chronic medical condition; active smoking, alcohol use or illicit drugs; current/recent treatment for OSAH; restless legs syndrome; shift work; travel across time zones in the past month or other active medical sleep disorder. The study was approved by our institutional Research Ethics Board and all participants gave written informed consent.

Potential participants underwent a home level II polysomnography (PSG) to screen for OSAH, which was defined by an apnea-hypopnea index (AHI) ≥5 events/h using AASM research (Chicago) scoring criteria (American Academy of Sleep Medicine Task Force, 1999). Those found to have OSAH then underwent measurement of arterial stiffness, immediately followed by 24-h ambulatory BP monitoring on the same day.

### Sleep Measurements

One-night home level II PSG was performed using the Embletta MPR with ST-proxy sleep system (Natus Inc., Mississauga, ON, United States) using our standard techniques (Champagne et al., 2009; Pamidi et al., 2014). A trained sleep technologist installed the recording equipment in the participant’s home. Sleep-wake state, arousals, and periodic limb movements were scored using AASM criteria (Berry et al., 2012), but respiratory events were scored using AASM research (Chicago) criteria (American Academy of Sleep Medicine Task Force, 1999) by a single experienced Registered Polysomnographic Technologist-certified scorer. We applied Chicago scoring criteria as they are more sensitive than AASM 2012 criteria, in that OSAH in pregnancy is characterized predominantly by hypopneas associated with microarousals rather than apneas or frequent desaturations (Champagne et al., 2009; Pamidi et al., 2014).

Participants also completed questionnaires assessing subjective sleep quality [Pittsburgh sleep quality index (PSQI)] (Buysse et al., 1989) and daytime sleepiness [Epworth sleepiness score (ESS)] (Johns, 1991).

### Blood Pressure and Heart Rate Measurements

Twenty-four-hour BP and heart rate were measured every 30 min during the daytime (6.00 AM–11.00 PM) and every 1 h at night (11.00 PM–6.00 AM) (Parati et al., 2014) using a validated Spacelabs Inc. device. Mean and standard deviation (SD) were calculated for both BP and heart rate. Heart rate variability was assessed via the coefficient of variability (CoV)–calculated as SD of heart rate divided by mean heart rate X 100. Office BP also was measured using a validated automated device BpTRU (average of last 5 of 6 unattended measurements obtained 1 min apart in the sitting or left lateral decubitus position) (Myers et al., 2014; Magee et al., 2015). In previous studies, BpTRU in non-pregnancy yielded values closely comparable to daytime values from 24-h ambulatory blood pressure monitoring (ABPM) (Myers et al., 2014).

### Arterial Stiffness Measurements

Participants underwent arterial stiffness measurement using Applanation tonometry (SphygmoCor; AtCor Medical, Sydney, NSW, Australia) to obtain measurements of cfPWV (central stiffness, main arterial stiffness outcome measure), carotid-radial PWV (crPWV) (peripheral stiffness), wave reflection assessed by AIx, and AIx corrected to a heart rate of 75 bpm (AIx75), as well as central BPs, pulse pressure amplification (PPA) (a measure of the progressive increase in pulse pressure from central to peripheral arteries), and subendocardial viability ratio (SEVR) (an index of myocardial oxygen supply and demand) using our established protocol (Gomez et al., 2016).

### Statistical Analysis

Based on BP dipping status on 24-h ABPM measurement, the participants were divided into dippers and non-dippers. The % BP dip was calculated as the difference between daytime and nighttime mean systolic blood pressure (SBP) divided by mean daytime SBP. Positive dipping (dippers) was defined as nocturnal SBP dip ≥ 10%, while <10% dip was defined as non-dipping. We similarly assessed ≥10% dipping for diastolic (DBP) and mean arterial pressure (MAP) (O’Brien et al., 2013; Parati et al., 2014).

Extreme dipping was defined as nocturnal BP dip ≥ 20% and inverse dipping was defined as any increase in BP overnight (Parati et al., 2014). Due to very small numbers (Figure 2A), extreme dippers were included in the dipper group and reverse dippers in the non-dipper group. Comparison of 24-h and office BP measures, as well as arterial stiffness measures between dipper and non-dipper groups was performed using unpaired *T*-tests. The primary analysis was to evaluate the relationship of OSAH severity to 24-h BP, in particular % nocturnal BP dipping. Linear regression was performed to evaluate the relationship between measures of OSAH severity [AHI; AHI during REM-sleep (REM-AHI); 4% oxygen desaturation index (4%ODI); % time of SpO2 below 90%] and mean 24-h BP. A separate linear regression model was constructed to evaluate the association between OSAH severity measures and % BP dipping. We adjusted for five potential confounding factors, including age, body mass index, gestational age at enrolment, parity, and number of antihypertensive medications in subsequent multiple regression models. For analysis of arterial stiffness, given uncertainty regarding normal arterial stiffness values in pregnancy, participants were divided into high vs. low arterial stiffness using the group median value for cfPWV of 7.2 m/s. Participant characteristics were then compared between high vs. low arterial stiffness groups. The relationship between OSAH severity measures and cfPWV as well as other arterial stiffness measures was also examined using linear regression models. All analyses were carried out using SPSS software, version 27.

## Results

### Study Participants

**FIGURE 1:**
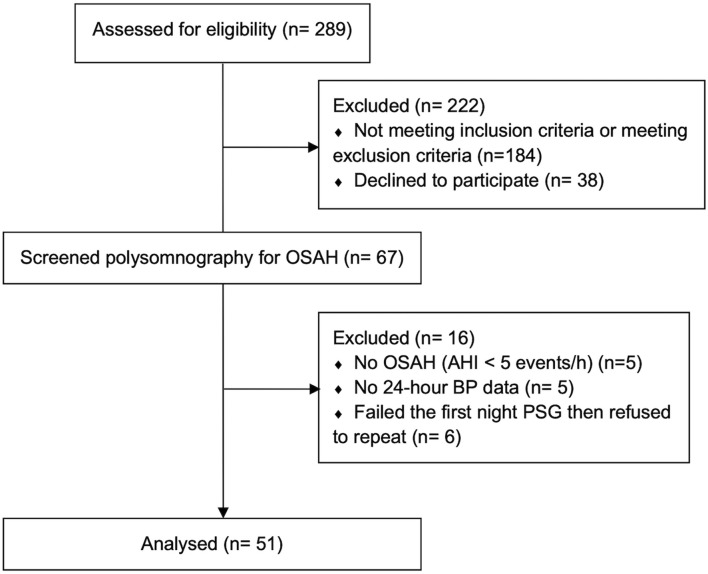
Enrollment flow diagram.

Two-hundred and eighty nine potential participants were identified from our obstetrics clinics between November 2017 and January 2021 (Figure 1). Of these, 38 women declined to participate in the study, 184 women were ineligible on the basis of no hypertension at screening, preterm delivery expected within 2 weeks, twin pregnancy, current CPAP treatment, medical co-morbidities, substance use, language barrier, or follow-up at another institution.

There were 67 potentially eligible participants who underwent PSG testing. 16 participants were excluded (five participants with AHI <5 events/h, five participants without 24-h BP data, and six participants failed the first night PSG then refused to repeat). The characteristics of the 51 HDP participants with OSAH who completed the study measurements are shown in Tables 1, 2. Participants were of mean age 36.5 ± 4.9 years, most were obese based on pre-pregnancy BMI and nearly half were Caucasian. The majority of participants were classified as having chronic hypertension (80.4%) and were receiving a single anti-hypertensive medication (64.7%) as well aspirin (82.4%) to reduce pre-eclampsia risk. A minority of participants had gestational diabetes. There was a history of pre-eclampsia in a previous pregnancy in 27.5%. PSG, 24-h ABPM, and arterial stiffness measurements were performed upon recruitment in either the second or third trimester. Most women were diagnosed with mild to moderate obstructive sleep apnea (Table 2). On average, they reported no or mild excessive daytime sleepiness based on ESS, but overall poor subjective sleep quality based on the PSQI (Table 1).

**TABLE 1:** Patient characteristics stratified by blood pressure dipping status.

	Total (*n* = 51)	Dippers (*n* = 11)	Non-dippers (*n* = 40)
**Maternal Age, years**	36.5 ± 4.9	37.7 ± 4.8	36.2 ± 4.9
**Pre-pregnancy BMI, kg/m** ^2^	34.6 ± 8.4	37.0 ± 6.5	33.9 ± 8.8
**BMI at enrolment, kg/m** ^2^	36.9 ± 8.6	38.4 ± 5.2	36.6 ± 9.4
**Gestational weight gain, kg**	5.7 ± 5.8	5.3 ± 7.7	5.9 ± 5.3
**GA at BP assessment, weeks**	27.6 ± 4.9	28.1 ± 3.9	27.5 ± 5.1
**GA at sleep study, weeks**	25.0 ± 4.9	25.3 ± 3.8	24.9 ± 5.2
**ESS**	9.9 ± 3.7	9.5 ± 3.9	10.0 ± 3.7
**PSQI**	8.9 ± 3.6	8.0 ± 3.5	9.3 ± 3.6
**Parity**			
Nulliparous	18 (35.3%)	4 (36.4%)	14 (35.0%)
Multiparous	33 (64.7%)	7 (63.6%)	26 (65.0%)
**Category of hypertension**			
Chronic hypertension	41 (80.4%)	9 (81.8%)	32 (80.0%)
Gestational hypertension	10 (19.6%)	2 (18.2%)	8 (20.0%)
**Anti-hypertensive medications:**			
Hydralazine	1 (2.0%)	0	1 (2.5%)
Methyldopa	12 (23.5%)	1 (9.1%)	11 (27.5%)
Labetalol	35 (68.6%)	8 (72.7%)	27 (67.5%)
Nifedipine	24 (47.1%)	4 (36.4%)	20 (50.0%)
Enalapril	1 (2.0%)	0	1 (2.5%)
**Other Medications:**			
ASA	42 (82.4%)	11 (100%)	31 (77.5%)
Insulin	8 (15.7%)	1 (9.1%)	7 (17.5%)
Metformin	5 (9.8%)	1 (9.1%)	4 (10.0%)
**Number of BP medications**			
1	33 (64.7%)	9 (81.8%)	24 (60.0%)
2	15 (29.4%)	2 (18.2%)	13 (32.5%)
≥3	3 (5.9%)	0	3 (7.5%)
**Diabetes status**			
Gestational diabetes	10 (19.6%)	1 (9.1%)	9 (22.5%)
Pre-existing diabetes	5 (9.8%)	1 (9.1%)	4 (10.0%)
**Ethnicity**			
Caucasian	23 (45.1%)	6 (54.5%)	17 (48.6%)
African American	10 (19.6%)	2 (18.2%)	8 (22.9%)
Other	18 (35.3%)	3 (27.3%)	15 (37.5%)
**Previous pregnancy with PrE**	14 (27.5%)	3 (27.3%)	11 (27.5%)
**Family history of CVD**	41 (80.4%)	9 (81.8%)	32 (80.0%)
**Severity of OSA**			
Mild	17 (33.3%)	4 (36.4%)	13 (32.5%)
Moderate	22 (43.1%)	4 (36.4%)	18 (45.0%)
Severe	12 (23.5%)	3 (27.3%)	9 (22.5%)

*Values are presented in means ± SD or numbers (%). BMI, body mass index; GA, gestational age; BP, blood pressure; ESS, Epworth Sleepiness Score; PSQI, Pittsburgh sleep quality index; PrE, pre-eclampsia; CVD, cardiovascular disease; OSA, obstructive sleep apnea.*

**TABLE 2:** Polysomnography characteristics of participants stratified by blood pressure dipping status.

	Total (*n* = 51)	Dippers (*n* = 11)	Non-dippers (*n* = 40)
Sleep efficiency, %	79.8 ± 13.7	76.8 ± 14.7	80.7 ± 13.5
Total sleep time, h	6.1 ± 1.6	5.6 ± 2.1	6.2 ± 1.5
Sleep stage:			
%NREM 1	19.2 ± 10.6	19.4 ± 10.1	19.1 ± 10.9
%NREM 2	47.1 ± 9.1	47.3 ± 6.7	47.1 ± 9.8
%NREM 3	16.8 ± 11.4	17.7 ± 12.5	16.5 ± 11.2
%REM	16.9 ± 7.6	15.7 ± 8.1	17.2 ± 7.6
Sleep onset latency, min	16.2 ± 25.9	9.8 ± 11.5	18.0 ± 28.6
WASO, min	72.1 ± 56.9	76.8 ± 50.0	70.7 ± 59.3
REM latency, min	100.5 ± 53.7	117.3 ± 68.3	95.4 ± 48.6
Microarousal index, /h	40.5 ± 19.7	41.3 ± 17.9	40.2 ± 20.4
Total sleep time in supine, %	20.0 ± 23.3	13.8 ± 14.4	21.8 ± 25.1
AHI, events/h	27.7 ± 26.4	25.3 ± 22.3	28.3 ± 27.6
AI, events/h	2.6 ± 7.6	1.3 ± 2.7	3.0 ± 8.5
HI, events/h	25.1 ± 21.0	24.0 ± 20.0	25.3 ± 21.6
REM-AHI, events/h	42.6 ± 27.3	47.8 ± 35.1	41.0 ± 24.8
NREM-AHI, events/h	22.7 ± 26.5	22.9 ± 22.6	22.7 ± 27.8
Supine-AHI, events/h	35.3 ± 26.2	36.2 ± 24.0	35.1 ± 27.2
Non-supine AHI, events/h	26.1 ± 29.6	24.2 ± 22.4	26.6 ± 31.6
Respiratory arousal index, events/h	20.1 ± 19.1	19.8 ± 21.2	20.2 ± 18.8
4% ODI, events/h	9.9 ± 22.2	5.2 ± 6.3	11.1 ± 24.5
Mean SpO_2_, %	95.7 ± 1.8	95.9 ± 1.8	95.7 ± 1.9
Nadir SpO_2_, %	87.3 ± 6.5	85.9 ± 7.9	87.7 ± 6.2
TST < 90%, %	2.0 ± 7.9	0.6 ± 1.5	2.4 ± 8.8

*Values are presented in means ± SD. WASO, wake after sleep onset; REM, rapid eye movement; NREM, non-rapid eye movement; AHI, apnea-hypopnea index; AI, apnea index; HI, hypopnea index; REM-AHI, apnea-hypopnea index during REM sleep; NREM-AHI, apnea-hypopnea index during NREM sleep; Supine-AHI, apnea-hypopnea index during supine position; Non-supine AHI, apnea-hypopnea index during non-supine position; 4%ODI, 4% oxygen desaturation index; SpO_2_, oxygen saturation during sleep; TST < 90%, total sleep time with oxygen saturation < 90%.*

The cohort was divided into dippers and non-dippers based on systolic BP dipping status. BP non-dipping was observed 40 participants (78.4%). No differences in demographics, subjective daytime sleepiness (ESS) or sleep quality (PSQI) or polysomnographic variables assessing sleep quality or fragmentation or OSAH severity were found between dippers and non-dippers (Tables 1, 2). We also found no significant relationships between % BP dipping and subjective sleepiness or sleep quality or with polysomnographic characteristics reflecting sleep quality and continuity (Supplementary Table 1).

On average, most participants had normal 24-h BP and office BP. As expected, nighttime systolic and diastolic BP was significantly higher in non-dippers than dippers, but there were no other significant differences in 24-h BP or in office BP between dippers and non-dippers (Table 3). Office BP measurements showed a significant positive correlation with the systolic and diastolic values obtained by 24-h ABPM (β = 0.75 and 0.70 for systolic and diastolic BP, respectively; *p*-value < 0.01). The mean % systolic BP dip in the dippers was 13.7 ± 3.4% vs. 4.1 ± 3.5% for non-dippers. Similarly, the mean % diastolic BP and MAP dip in the dippers and non-dippers were 16.6 ± 5.2% vs. 4.4 ± 5% for diastolic BP dip, and 15.2 ± 4.4% vs. 4.7 ± 4.2% for MAP dip, respectively (Table 3).

**TABLE 3:** 24-h blood pressure and % blood pressure dipping of participants stratified by blood pressure dipping status.

	Total (*n* = 51)	Dippers (*n* = 11)	Non-dippers (*n* = 40)	*p*-value
**24-h blood pressure (mm Hg)**				
24-h systolic blood pressure	122.3 ± 10.3	119.0 ± 13.1	123.3 ± 9.4	0.23
24-h diastolic blood pressure	71.7 ± 9.2	68.6 ± 10.2	72.5 ± 8.8	0.20
24-h mean arterial pressure	88.5 ± 9.1	85.2 ± 10.6	89.4 ± 8.5	0.17
Daytime systolic blood pressure	124.5 ± 10.1	123.1 ± 13.0	124.8 ± 9.3	0.62
Daytime diastolic blood pressure	73.5 ± 9.3	71.6 ± 9.8	74.0 ± 9.2	0.46
Daytime mean arterial pressure	90.5 ± 8.9	89.1 ± 10.2	91.0 ± 8.6	0.55
Nighttime systolic blood pressure*	116.8 ± 11.7	106.2 ± 11.5	119.7 ± 10.0	0.00
Nighttime diastolic blood pressure*	66.5 ± 9.4	58.1 ± 8.2	68.8 ± 8.4	0.00
Nighttime mean arterial pressure*	82.9 ± 9.9	73.9 ± 9.1	85.4 ± 8.7	0.00
**% Dipping of blood pressure**				
Systolic blood pressure	6.2 ± 5.3	13.7 ± 3.4	4.1 ± 3.5	0.00
Diastolic blood pressure	9.4 ± 7.9	16.6 ± 5.2	4.4 ± 5.0	0.00
Mean arterial pressure	8.4 ± 6.6	15.2 ± 4.4	4.7 ± 4.2	0.00
**Office blood pressure by BpTRU (mm Hg)**				
Systolic blood pressure	119.6 ± 11.1	120.8 ± 13.8	119.3 ± 10.4	0.70
Diastolic blood pressure	80.1 ± 8.7	78.8 ± 12.3	80.4 ± 7.6	0.60
Mean arterial pressure	92.2 ± 9.5	91.1 ± 13.1	92.5 ± 8.5	0.74

*p-value < 0.01 for dippers vs. non-dippers.

*Values are presented in means ± SD. % Dipping of blood pressure = 100 × (daytime-nighttime blood pressure)/daytime blood pressure.*

### Association Between Obstructive Sleep Apnea-Hypopnea Severity and 24-H Blood Pressure Measures

**FIGURE 2:**
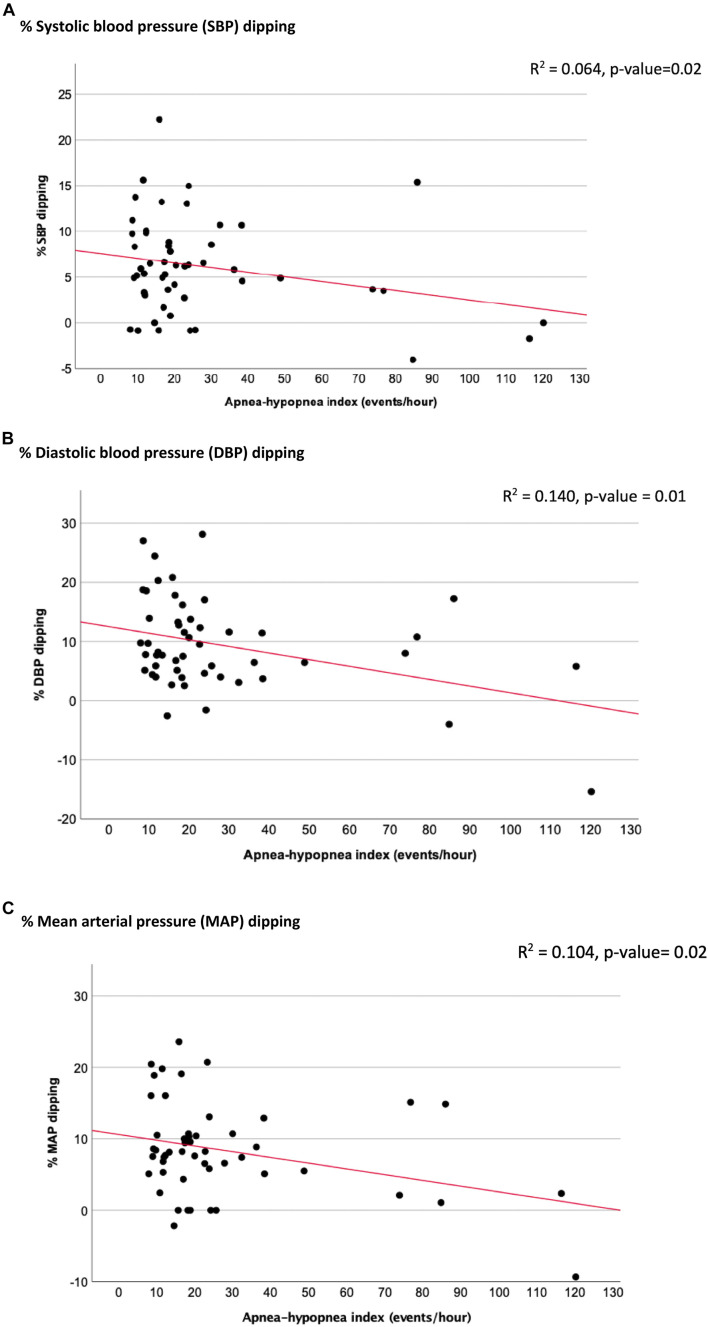
Linear regression models demonstrated association between % blood pressure dipping and apnea-hypopnea index. **(A)** % Systolic blood pressure (SBP) dipping. **(B)** % Diastolic blood pressure (DBP) dipping. **(C)** % Mean arterial pressure (MAP) dipping.

No significant relationships were found between AHI or other OSAH severity measures and 24-h SBP, DBP, or MAP values in regression analyses (Table 4). However, AHI showed a significant inverse correlation with % SBP dip following adjustment for age, body mass index, parity, gestational age, and BP medications (β = −0.11, 95% CI = −0.20 to −0.02; *p* = 0.02) (Table 5 and Figure 2). Similarly, significant inverse correlations were observed between AHI and % DBP dip (β = −0.16, 95% CI = −0.29 to −0.04; *p* = 0.01) and % MAP dip (β = −0.13, 95% CI = −0.24 to −0.02; *p* = 0.02) (Table 5 and Figure 2). As shown in Supplementary Tables 2, 3, we found no significant relationships between participants demographics and 24-h BP or % BP dip in regression analyses.

**TABLE 4:** Regression models for association between 24-h blood pressures and obstructive sleep apnea severity measures.

	24-h SBP		24-h DBP		24-h MAP	
	Unadjusted β (95% CI)	Adjusted* β (95% CI)	Unadjusted β (95% CI)	Adjusted* β (95% CI)	Unadjusted β (95% CI)	Adjusted* β (95% CI)
AHI	−0.03(−0.14,0.08)	−0.09(−0.25,0.08)	−0.09(−0.19,0.00)	−0.01(−0.14,0.13)	−0.06(−0.16,0.04)	−0.02(−0.16,0.12)
REM-AHI	0.06(−0.06,0.17)	0.06(−0.08,0.21)	−0.08(−0.18,0.02)	−0.02(−0.14,0.11)	−0.04(−0.14,0.07)	0.00(−0.12,0.13)
NREM-AHI	−0.05(−0.17,0.06)	−0.14(−0.31,0.03)	−0.12(−0.21,−0.02)	−0.06(−0.20,0.08)	−0.09(−0.19,0.01)	−0.07(−0.22,0.08)
4% ODI	0.02(−0.11,0.15)	0.03(−0.14,0.19)	−0.07(−0.18,0.05)	0.06(−0.08,0.20)	−0.03(−0.15,0.08)	0.05(−0.01,0.19)
Resp arousal index	−0.07(−0.23,0.09)	−0.16(−0.37,0.05)	−0.15(−0.29,−0.01)	−0.08(−0.25,0.10)	−0.11(−0.25,0.03)	−0.08(−0.26,0.10)
Nadir SpO_2_	−0.08(−0.53,0.37)	−0.00(−0.56,0.56)	0.21(−0.18,0.60)	−0.02(−0.49,0.45)	0.13(−0.26,0.52)	0.03(−0.45,0.50)
TST < 90%	0.02(−0.35,0.38)	0.03(−0.43,0.49)	−0.31(−0.62,−0.00)	−0.07(−0.45,0.32)	−0.20(−0.53,0.11)	−0.06(−0.45,0.33)
Microarousal index	−0.03(−0.19,0.13)	−0.09(−0.31,0.13)	−0.14(−0.27,−0.01)	−0.08(−0.26,0.11)	−0.09(−0.22,0.05)	−0.06(−0.25,0.12)
Total sleep time	−0.62(−2.46,1.22)	−0.31(−2.13,1.51)	0.21(−1.43,1.85)	0.32(−1.21,1.84)	−0.17(−1.80,1.50)	0.03(−1.51,1.58)

*Adjusted with age, body mass index at enrolment, parity, number of BP medications, gestational age at blood pressure assessment.

*SBP, systolic blood pressure; DBP, diastolic blood pressure; MAP, mean arterial pressure; AHI, apnea-hypopnea index; REM-AHI, apnea-hypopnea index during REM sleep; NREM-AHI, apnea-hypopnea index during NREM sleep; 4%ODI, 4% oxygen desaturation index; Resp arousal index, respiratory arousal index; SpO_2_, oxygen saturation during sleep; TST < 90%, total sleep time with oxygen saturation < 90%.*

**TABLE 5:** Regression models for association between % blood pressure dipping and obstructive sleep apnea severity measures.

	%SBP dip		%DBP dip		%MAP dip	
	Unadjusted β (95% CI)	Adjusted* β (95% CI)	Unadjusted β (95% CI)	Adjusted* β (95% CI)	Unadjusted β (95% CI)	Adjusted* β (95% CI)
AHI	−0.05 (−0.10, 0.01)	−**0.11 (**−**0.20,**−**0.02)**	−**0.11 (**−**0.19,**−**0.03)**	−**0.16 (**−**0.29,**−**0.04)**	−**0.08 (**−**0.15,**−**0.01)**	−**0.13 (**−**0.24,**−**0.02)**
REM-AHI	−0.00 (−0.10, 0.10)	−0.03 (−0.10, 0.10)	−0.06 (−0.15, 0.03)	−0.05 (−0.17, 0.07)	−0.05 (−0.12, 0.03)	−0.05 (−0.15, 0.05)
NREM-AHI	−0.03 (−0.10, 0.03)	−0.08 (−0.20, 0.02)	−0.09 (−0.17, −0.01)	−0.13 (−0.26, 0.01)	−0.07 (−0.14, 0.01)	−0.11 (−0.22, 0.01)
4% ODI	−**0.08 (**−**0.15,**−**0.02)**	−**0.15 (**−**0.23,**−**0.06)**	−**0.17 (**−**0.26,**−**0.09)**	−**0.23 (**−**0.35,**−**0.11)**	−**0.13 (**−**0.21,**−**0.06)**	−**0.18 (**−**0.30,**−**0.09)**
Resp arousal index	−0.03 (−0.11, 0.05)	−0.06 (−0.18, 0.05)	−0.09 (−0.21, 0.03)	−0.08 (−0.25, 0.09)	−0.06 (−0.16, 0.04)	−0.06 (−0.21, 0.09)
Nadir SpO_2_	0.08 (−0.15, 0.31)	0.11 (−0.21, 0.43)	**0.44 (0.13, 0.76)**	**0.56 (0.13, 0.99)**	**0.30 (0.03, 0.58)**	0.38 (0.00, 0.76)
TST < 90%	−**0.19 (**−**0.37, 0.00)**	−**0.32 (**−**0.57,**−**0.07)**	−**0.50 (**−**0.74,**−**0.26)**	−**0.67 (**−**0.99,**−**0.34)**	−**0.37 (**−**0.58,**−**0.16)**	−**0.54 (**−**0.82,**−**0.26)**
Microarousal index	−0.02 (−0.10, 0.05)	−0.05 (−0.17, 0.08)	−0.09 (−0.20, 0.02)	−0.09 (−0.27, 0.08)	−0.06 (−0.16, 0.04)	−0.06 (−0.22, 0.09)
Total sleep time	0.05 (−0.90, 0.99)	0.00 (−1.04, 1.05)	0.05 (−1.36, 1.46)	−0.01 (−1.51, 1.49)	−0.28 (−1.46, 0.90)	−0.40 (−1.66, 0.87)

*Adjusted with age, body mass index at enrolment, parity, number of BP medications, gestational age at blood pressure assessment.

*% Blood pressure dip = 100 × (daytime-nighttime blood pressure)/daytime blood pressure. SBP, systolic blood pressure; DBP, diastolic blood pressure; MAP, mean arterial pressure, AHI, apnea-hypopnea index; REM-AHI, apnea-hypopnea index during REM sleep; NREM-AHI, apnea-hypopnea index during NREM sleep; 4% ODI, 4% oxygen desaturation index; Resp arousal index, respiratory arousal index; SpO_2_, oxygen saturation during sleep; TST < 90%, total sleep time with oxygen saturation < 90%.*

*Bold values means p-value < 0.05.*

In addition, 4% oxygen desaturation index showed a significant inverse correlation with % SBP, DBP and MAP dip in adjusted regression analyses (β = −0.15, 95% CI = −0.23 to −0.06; *p* < 0.01 for % SBP dip, β = −0.23, 95% CI = −0.35 to −0.11; *p* < 0.01 for % DBP dip and β = −0.18, 95% CI = −0.30 to −0.09; *p* < 0.01 for % MAP dip). Moreover, there were significant positive correlations between total sleep time with oxygen saturation <90% and % SBP, DBP and MAP dip in adjusted regression analyses (β = −0.32, 95% CI = −0.57 to −0.07; *p* = 0.01 for % SBP dip, β = −0.67, 95% CI = −0.99 to −0.34; *p* < 0.01 for % DBP dip and β = −0.54, 95% CI = −0.82 to −0.26; *p* < 0.01 for % MAP dip). We also found a positive significant correlation between nadir oxygen saturation and % DBP dip in adjusted regression analysis (β = 0.56, 95% CI = 0.13 to 0.99; *p* = 0.01) and % MAP dip in unadjusted regression analysis (β = 0.30, 95% CI = 0.03 to 0.58; *p* = 0.03) but not % SBP dip (Table 5).

### Association Between Obstructive Sleep Apnea-Hypopnea Severity and 24-H Heart Rate Measures

There were no significant differences in 24-h, daytime and nighttime heart rate between dippers and non-dippers stratified by SBP, DBP, or MAP dipping status (Supplementary Table 4).

The nocturnal decline in heart rate was small in all groups and did not differ according to dipping status. We also found no significant differences in heart rate variability (CoV) between dippers and non-dippers for 24 h, daytime or nighttime (Supplementary Table 4). No significant relationships were found between AHI or other OSAH severity measures and 24-h, daytime, or nighttime heart rate CoV (Supplementary Table 5).

### Association Between Obstructive Sleep Apnea-Hypopnea Severity and Arterial Stiffness Measures

Of the 43 patients who completed arterial stiffness measurement, there were 11 dippers and 32 non-dippers. The mean cfPWV was 7.4 ± 1.2 m/s. The participants were divided into high vs. low central arterial stiffness according to the group median cfPWV at 7.2 m/s. No differences between sleep characteristics for high and low cfPWV groups were observed (Supplementary Table 6).

There was no significant difference in the proportion of high cfPWV patients between dippers and non-dippers. Likewise, there was no significant difference in other arterial stiffness measures between dippers and non-dippers except for a higher SEVR with non-dippers (Table 6).

**TABLE 6:** Arterial stiffness characteristics of participants stratified by blood pressure dipping status.

	Total (*n* = 43)	Dippers (*n* = 11)	Non-dippers (*n* = 32)	*p*-value
Patients who had high cfPWV (≥7.2 m/s)	22 (51.2%)	7 (63.6%)	15 (46.9%)	0.34
Carotid femoral pulse wave velocity, m/s	7.4 ± 1.2	7.8 ± 1.2	7.2 ± 1.1	0.18
Carotid radial pulse wave velocity, m/s	8.2 ± 1.2	8.3 ± 1.6	8.1 ± 1.1	0.77
Carotid radial/femoral PWV	1.1 ± 0.2	1.1 ± 0.2	1.2 ± 0.3	0.40
Augmentation index, %	14.0 ± 9.6	14.5 ± 7.8	13.9 ± 10.2	0.87
Corrected augmentation index with HR 75 bpm, %	17.7 ± 8.7	19.3 ± 7.5	17.2 ± 9.1	0.51
Subendocardial viability ratio, %	119.9 ± 18.3	109.6 ± 19.1	123.4 ± 16.9	0.03
Pulse pressure amplification, mm Hg	1.5 ± 0.2	1.5 ± 0.1	1.5 ± 0.2	0.81
Central SBP, mm Hg	98.3 ± 20.2	88.8 ± 29.7	101.5 ± 15.1	0.07
Central DBP, mm Hg	75.8 ± 8.9	74.1 ± 10.5	76.3 ± 8.4	0.49
Peripheral SBP, mm Hg	115.1 ± 12.0	114.2 ± 11.3	115.5 ± 12.5	0.77
Peripheral DBP, mm Hg	73.6 ± 8.6	70.7 ± 9.9	74.6 ± 8.0	0.21

*Values are presented in means ± SD or numbers (%). cfPWV, carotid femoral pulse wave velocity; PWV, pulse wave velocity; HR, heart rate; SBP, systolic blood pressure; DBP, diastolic blood pressure.*

**FIGURE 3:**
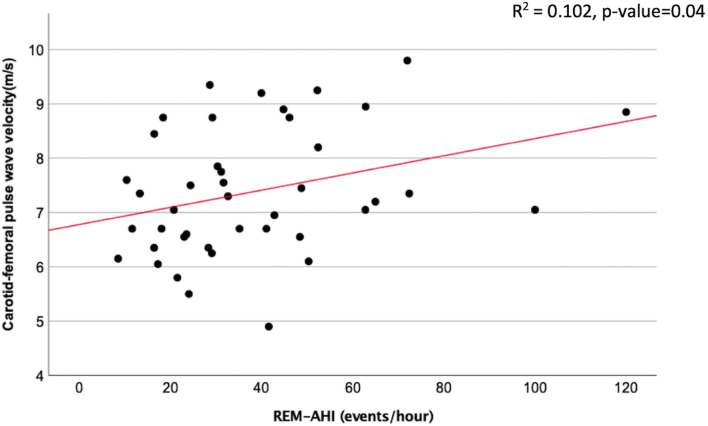
Linear regression model demonstrated association between carotid femoral pulse wave velocity and REM-apnea hypopnea index (REM-AHI).

There were significant relationships between cfPWV and BMI in unadjusted and adjusted regression analyses [β = 0.06 (95% CI = 0.01 to 0.11) and 0.08 (95% CI = 0.02 to 0.13); *p* = 0.02 and 0.01, respectively] (Table 7 and Supplementary Figure 1). Similarly, a significant positive association between cfPWV and maternal age was observed in adjusted regression analysis. While most measures of OSAH severity did not show significant associations with arterial stiffness measures, we did observe a significant positive correlation between cfPWV and the AHI during REM sleep (REM-AHI) in unadjusted regression analysis (β = 0.02, 95% CI = 0.001 to 0.03; *p* = 0.04). However, this was no longer significant following adjustment for age, body mass index, gestational age, and parity (β = 0.01, 95% CI = −0.01 to 0.03; *p* = 0.23) (Table 7 and Figure 3). There were no significant correlations between cfPWV and other OSAH severity measures (Table 7).

**TABLE 7:** Regression models for association between carotid femoral pulse wave velocity and demographics and obstructive sleep apnea severity measures.

	Carotid femoral pulse wave velocity		
	Unadjusted β (95% CI)	Adjusted β* (95% CI)	Adjusted β** (95% CI)
Age	0.04(−0.03,0.01)	**0.07 (0.002, 0.14)**	**0.07 (0.002, 0.14)**
BMI at enrolment	**0.06 (0.01, 0.11)**	**0.08 (0.02, 0.13)**	**0.08 (0.02, 0.13)**
Gestational age at BP assessment	−0.01(−0.09,0.07)	−0.01(−0.08,0.07)	−0.01(−0.08,0.07)
Parity	0.18(−0.17,0.54)	0.20(−0.14,0.54)	0.20(−0.14,0.54)
AHI	0.01(−0.02,0.04)	0.01(−0.02,0.04)	−0.01(−0.04,0.02)
REM-AHI	**0.02 (0.001, 0.03)**	**0.02 (0.002, 0.03)**	0.01(−0.01,0.03)
NREM-AHI	0.01(−0.02,0.03)	0.01(−0.02,0.03)	−0.01(−0.04,0.02)
4% ODI	−0.02(−0.09,0.05)	−0.01(−0.08,0.06)	−0.03(−0.10,0.04)
Respiratory arousal index	0.01(−0.02,0.04)	0.01(−0.02,0.04)	−0.00(−0.03,0.03)
Mean SpO_2_	0.01(−0.26,0.28)	−0.04(−0.32,0.25)	0.06(−0.21,0.33)
Nadir SpO_2_	−0.00(−0.08,0.07)	−0.02(−0.10,0.06)	0.03(−0.05,0.11)
TST < 90%	−0.26(−0.71,0.19)	−0.21(−0.70,0.28)	−0.38(−0.83,0.07)

*Adjusted with age, parity, gestational age at BP assessment.

**Adjusted with age, parity, gestational age at BP assessment and BMI at enrolment.

*BMI, body mass index; AHI, apnea-hypopnea index; REM-AHI, apnea-hypopnea index during REM sleep; NREM-AHI, apnea-hypopnea index during NREM sleep; 4% ODI, 4% oxygen desaturation index; SpO_2_, oxygen saturation during sleep; TST < 90%, total sleep time with oxygen saturation < 90%.*

*Bold values means p-value < 0.05.*

Significant inverse associations were observed between cfPWV and 24-h BP values in adjusted regression analyses, but cfPWV was not correlated with % nocturnal BP dipping (Supplementary Table 7).

## Discussion

Pre-eclampsia affects 2–8% of pregnancies and remains a leading cause of maternal and neonatal mortality (Shah and Gupta, 2019). While previous studies have identified risk factors for progression to pre-eclampsia among women with gestational hypertension, (Florio et al., 2005; Davis et al., 2007; Bellomo et al., 2011; Rana et al., 2012; Melamed et al., 2014; Phan et al., 2021) there is a need to identify risk factors which can be modified to improve adverse maternal and fetal outcomes in women with HDP.

Estimates of the prevalence of OSAH in pregnancy by the third trimester range from 17 to 45% (Pamidi and Kimoff, 2018). Several studies have shown that OSAH is strongly associated with a greater risk of HDP (Champagne et al., 2009; Ding et al., 2014; Pamidi et al., 2014; Bourjeily et al., 2017; Li et al., 2018; Liu et al., 2019; Querejeta Roca et al., 2020; Lu et al., 2021). In keeping with this, in the present study, out of 67 HDP women who initially met eligibility criteria, 56 (83.6%) tested positive for OSAH.

While the high prevalence of OSAH in our pregnant participants with chronic and gestational hypertension supports the link between OSAH and HDP, we did not observe any significant relationships between OSAH severity measures and 24-h BP values. We believe the most likely explanation for the lack of an association between OSAH severity measures and 24-h BP profile is that our participants were on at least one anti-hypertensive medication and were followed regularly by our maternal-fetal medicine specialists with close monitoring of BP control. BP values overall were within the target normal range so that a relationship between OSAH severity and BP level was not evident.

It is well known that a non-dipping BP pattern predicts poor cardiovascular outcomes in non-pregnant patients with hypertension (de la Sierra et al., 2014; Seif et al., 2014; Torres et al., 2015; Genta-Pereira et al., 2018; Crinion et al., 2019; Cuspidi et al., 2019; Pio-Abreu et al., 2021). In HDP patients, BP non-dipping predicts preterm delivery, FGR, and poor maternal cardiac function (Ilic et al., 2017). Thus a nocturnal non-dipping BP pattern would appear to be an important measure for risk stratification in patients with HDP. We found a high proportion (nearly 80%) of BP non-dipping among our HDP patients with OSAH. Previous studies reported that blunting of circadian pattern was more common in pre-eclampsia patients compared to normotensive pregnant women (Oney and Meyer-Sabellek, 1990; Ayala et al., 1997; Hermida et al., 2000; Gupta et al., 2011). In pre-eclampsia patients, the prevalence of systolic and diastolic BP non-dipping was 40 and 65%, respectively, while in normotensive pregnant women, non-dippers were found only 23 and 34.3% for systolic and diastolic BP (Gupta et al., 2011).

Previous studies in the general population indicate that OSAH is associated with BP non-dipping (Seif et al., 2014; Torres et al., 2015; Genta-Pereira et al., 2018; Crinion et al., 2019; Cuspidi et al., 2019; Pio-Abreu et al., 2021). However, the present study is the first to demonstrate that increasing severity of OSAH as reflected by AHI, 4% ODI, and TST < 90%, is associated with decreased nocturnal BP dipping in hypertensive pregnant women. These associations remained significant after adjusting for age, body mass index, parity, gestational age, and BP medications. We found that each 1 event/h increase in AHI was associated with 0.11 and 0.16% reduction in % SBP and DBP dipping, respectively. Similarly, we found each 1 event/h increase in 4% ODI was associated with 0.15 and 0.23% reduction in % SBP and DBP dipping, respectively. The nocturnal hypertension in OSAH participants may be explained by the BP surge at the end of the obstructive respiratory events (Somers et al., 1995).

Recent meta-analyses demonstrate that CPAP treatment improves BP in OSAH with the largest changes being in nocturnal BP values, with restoration of dipping in some studies (Bratton et al., 2015; Patil et al., 2019). The link between OSAH and BP non-dipping in the present study therefore raises the possibility that treatment of OSAH could restore the nocturnal dipping pattern in HDP and thus potentially attenuate the risk for development of pre-eclampsia. The effects of CPAP treatment on 24 h BP are being explored in our ongoing pilot controlled trial (NCT03309826).

Previous studies have shown that arterial stiffness is elevated in patients with OSAH (Kohler et al., 2008; Doonan et al., 2011; Jenner et al., 2017) and one study demonstrated an association between OSAH, increased arterial stiffness and a non-dipping BP pattern in patients with hypertension (odd ratio = 3.03; 95% CI = 1.08–0.35) (Jenner et al., 2017). Evidence from meta-analyses demonstrated that CPAP treatment is associated with improved arterial stiffness in patients with OSAH, (Kohler et al., 2013; Vlachantoni et al., 2013; Wons and Kohler, 2015; Lin et al., 2016; Chalegre et al., 2020) while a recent RCT showed that 6-months CPAP treatment prevented progression of arterial stiffness in patients with moderate-severe OSAH and resistant hypertension (Cardoso et al., 2020).

There is increasing interest in the role of arterial stiffness in HDP (Vlachopoulos et al., 2010; Joyeux-Faure et al., 2018; Theorell-Haglöw et al., 2019). Meta-analysis has shown significant increases in arterial stiffness measurements in women with pre-eclampsia compared with those with gestational hypertension (Hausvater et al., 2012). Recently, we have shown a change point early in the second trimester in carotid femoral pulse wave velocity (cfPWV, the gold standard measure of arterial stiffness) trajectories measured throughout pregnancy in women destined to develop pre-eclampsia vs. those who did not (Phan et al., 2021). However, there has to date been no study on the relationship between OSAH and arterial stiffness in HDP patients.

In this study, we did not find significant relationships between overall AHI, oxygen desaturation, or sleep time with SpO_2_ below 90% and arterial stiffness measures, which contrasts with previous findings in non-pregnant OSAH (Theorell-Haglöw et al., 2019). This discrepancy may be explained in part by the variability of central arterial stiffness during pregnancy in the second to third trimester, particularly if they are destined to develop pre-eclampsia or not (Khalil et al., 2009). However, we did find a significant association between cfPWV and increasing severity of OSAH during REM sleep. Data from several cohort studies have indicated that REM-related OSA in particular is linked to incident hypertension and vascular risk (Mokhlesi et al., 2014; Aurora et al., 2018), which is consistent with the interaction we observed between REM OSAH and arterial stiffness. The significant correlation between REM AHI and cfPWV remained significant after adjustment for age, parity, and weeks of gestation, but not when additionally adjusted for BMI (Table 7). We also found significant relationships between cfPWV and BMI in unadjusted and adjusted regression analyses. This observation is consistent with previous findings in morbidly obese patients that increasing BMI was independently associated with higher cfPWV in women but not in men (Nordstrand et al., 2011). While one interpretation would be that the interaction of REM AHI with cfPWV is solely due to the confounding effect of obesity, the interaction is likely more complex in that obesity will tend to worsen OSAH severity (reduced upper airway caliber due to fat deposition and lung volume-dependent effects, reduced O2 stores with more events meeting desaturation criteria) and in that respect may be part of the mechanistic pathway by which OSAH may alter vascular function.

There are several strengths of our study. OSAH was diagnosed based on complete overnight PSG yielding greater diagnostic accuracy than limited-channel sleep recordings. More importantly, this is the first demonstration of a significant inverse relationship between measures of OSAH severity and 24-h BP or arterial stiffness in HDP patients.

There were some limitations in our study. In that 24-h BP monitoring was performed only after potential participants were determined to have OSAH, we did not have a non-OSAH comparison group. Furthermore, our sample size was limited thus constraining statistical power. While we were unable to demonstrate a significant association between a OSAH severity and 24-h BP, our patient cohort was being closely followed in obstetrical medicine, were all receiving at least one anti-hypertensive medication and BP was controlled and generally within the normal range. Ethical considerations precluded withdrawal of medication for the present study. However, future studies could prospectively evaluate women with a prior HDP no longer requiring medication post-partum but planning a subsequent pregnancy, to longitudinally assess the relationship of OSAH and BP control and nocturnal dipping prior to and over the course of pregnancy. While we found significant correlations in this study between AHI, ODI and % dipping, as well as between arterial stiffness and REM AHI, as shown in Figures 2, 3, these relationships appear to have been influenced by the smaller number of severe patients. These findings suggest that the impact of OSAH and potential benefits of OSAH treatment with respect to vascular risk reduction may be most marked in severe OSAH. However, this remains to be determined across the range of OSAH severity in our ongoing pilot RCT and other interventional studies. Finally, in this study we assessed the relationship between OSAH, BP values, non-dipping and arterial stiffness at a single time point relatively late in pregnancy. Our recent work indicates that in longitudinal follow-up from early to late pregnancy, changes in arterial stiffness may predict subsequent development of pre-eclampsia (Phan et al., 2021). Thus further work is warranted to evaluate the relationships between OSAH, BP profile and arterial stiffness longitudinally over the course of pregnancy and evaluate the association between these changes and maternal and fetal outcomes.

## Conclusion

In this cohort, we found BP non-dipping in a majority of women with HDP and OSAH. We observed a significant inverse relationship between OSAH severity and % nocturnal BP dipping. Furthermore, increased arterial stiffness was associated with increasing severity of OSAH during REM sleep in unadjusted although not adjusted analysis. These findings therefore raise the possibility that OSAH may represent a therapeutic target to improve BP profile and vascular function, and thus reduce risk, in HDP. Our ongoing pilot RCT will explore the impact of OSAH treatment on 24 h-BP, arterial stiffness, and other outcomes in HDP patients.

## Data Availability Statement

The raw data supporting the conclusions of this article will be made available by the authors, without undue reservation.

## Ethics Statement

The studies involving human participants were reviewed and approved by McGill University Health Centre Research Ethics Board. The patients/participants provided their written informed consent to participate in this study.

## Author Contributions

PP and NG conducted the study and gathered data in concert with KR, nurse research coordinator. SD provided arterial stiffness measurements. AO scored all PSG studies. PP analyzed the study data. AB provided statistical guidance and support. SP, SD, RG, and ND were co-investigators who contributed to study design, and interpretation. RJK was the principal investigator/editing author and assumes final responsibility for study integrity. All authors contributed to the article and approved the submitted version.

## Conflict of Interest

The authors declare that the research was conducted in the absence of any commercial or financial relationships that could be construed as a potential conflict of interest.

## Publisher’s Note

All claims expressed in this article are solely those of the authors and do not necessarily represent those of their affiliated organizations, or those of the publisher, the editors and the reviewers. Any product that may be evaluated in this article, or claim that may be made by its manufacturer, is not guaranteed or endorsed by the publisher.
