# Supplementary material for: Effect of Maternal Obstructive Sleep Apnea-Hypopnea on 24-Hour Blood Pressure, Nocturnal Blood Pressure Dipping and Arterial Stiffness in Hypertensive Disorders of Pregnancy
Source: Front Physiol. 2021 Oct 18;12:747106. doi: 10.3389/fphys.2021.747106 (PMC8558510; doi:10.3389/fphys.2021.747106)

# Supplementary Tables and Figure

**Table S1:**  Correlations between % blood pressure dipping and polysomnographic characteristics

|  | **% SBP dip**  **r (p-value)** | **% DBP dip**  **r (p-value)** | **% MAP dip**  **r (p-value)** |
| --- | --- | --- | --- |
| **ESS**  **PSQI**  **Sleep efficiency, %**  **Total sleep time, hours**  **Sleep onset latency, minutes**  **Wake after sleep onset, minutes**  **Microarousal index, events/hour**  **Respiratory arousal index, events/hour** | -0.136(0.36)  -0.081(0.60)  -0.052(0.72)  0.014(0.92)  -0.127(0.38)  0.001(0.99)  -0.089(0.55)  -0.121(0.41) | -0.215(0.14)  -0.284(0.06)  0.014(0.92)  0.011(0.94)  -0.180(0.22)  -0.084(0.57)  -0.234(0.11)  -0.225(0.12) | -0.236(0.11)  -0.200(0.19)  -0.065(0.66)  -0.069(0.64)  -0.142(0.33)  0.000(1.00)  -0.182(0.21)  -0.178(0.22) |

r = Pearson’s correlation coefficient

ESS: Epworth Sleepiness Score, PSQI: Pittsburgh sleep quality index

**Table S2:** Regression models for association between 24-hour blood pressures and demographics

|  | **24-hour SBP** | | **24-hour DBP** | | **24-hour MAP** | |
| --- | --- | --- | --- | --- | --- | --- |
|  | **Unadjusted**  **β**  **(95% CI)** | **Adjusted***  **β**  **(95% CI)** | **Unadjusted**  **β**  **(95% CI)** | **Adjusted***  **β**  **(95% CI)** | **Unadjusted**  **β**  **(95% CI)** | **Adjusted***  **β**  **(95% CI)** |
| **Age**  **BMI at enrolment**  **Weight gain**  **GA at BP assessment**  **Parity**  **Number BP meds**  **Gestational diabetes**  **ESS**  **PSQI**  **Previous PrE**  **Family history of CVD**  **Category of HTN** | -0.26(-0.86,0.34)  0.08(-0.26,0.42)  0.06(-0.48,0.59)  -0.26(-0.86,0.35)  -2.51(-5.36,0.34)  5.10(0.94,9.25)  -5.09(-9.28,-0.89)  0.29(-0.52,1.10)  -0.03(-0.96,0.90)  1.41(-5.14,7.96)  6.01(-1.16,13.18)  1.45(-5.91,8.81) | -0.43(-1.1,0.15)  0.01(-0.33,0.34)  0.19(-0.35,0.73)  -0.08(-0.68,0.52)  -2.48(-5.34,0.37)  5.45(1.28,9.62)  -3.60(-8.25,1.00)  0.21(-0.64,1.05)  -0.25(-1.21,0.71)  1.50(-5.03,8.02)  5.66(-1.40,12.72)  -0.02(-8.08,8.04) | -0.37(-0.90,0.15)  -0.29(-0.58,0.00)  -0.09(-0.54,0.35)  0.17(-0.37,0.70)  -1.69(-4.26,0.88)  3.64(-0.14,7.41)  -7.07(-10.46,-3.69)  0.15(-0.60,0.89)  -0.24(-1.05,0.56)  1.52(-4.30,7.33)  4.09(-2.36,10.54)  4.74(-1.67,11.15) | -0.62(-1.10,-0.13)  -0.35(-0.63,-0.08)  0.02(-0.42,0.46)  0.19(-0.31,0.69)  -2.39(-4.76,-0.01)  4.43(0.95,7.91)  -4.89(-8.57,-1.22)  0.18(-0.52,0.89)  -0.36(-1.13,0.42)  0.50(-4.95,5.95)  4.44(-1.47,10.35)  3.49(-3.15,10.13) | -0.32(-0.85,0.20)  -0.15(-0.45,0.15)  -0.01(-0.47,0.44)  0.03(-0.50,0.57)  -2.10(-4.53,0.51)  4.50(0.84,8.15)  -6.22(-9.69,-2.75)  0.16(-0.57,0.88)  -0.22(-1.03,0.60)  1.59(-4.16,7.34)  3.84(-2.55,10.23)  3.87(-2.52,10.26) | -0.54(-1.0,-0.05)  -0.21(-0.49,0.07)  0.10(-0.35,0.56)  0.13(-0.38,0.63)  -2.46(-0.49,-0.06)  5.13(1.61,8.66)  -4.17(-8.0,-0.36)  0.14(-0.58,0.86)  -0.40(-1.2,0.39)  0.91(-4.61,6.42)  3.83(-2.19,9.85)  2.57(-4.20,9.33) |

*Adjusted with age, BMI at enrolment, parity, number of BP medications, Gestational age at BP assessment.

SBP: systolic blood pressure, DBP: diastolic blood pressure, MAP: mean arterial pressure,

BMI: body mass index, Weight gain: gestational weight gain, GA: gestational age, BP: blood pressure, ESS: Epworth Sleepiness Score, PSQI: Pittsburgh sleep quality index, Previous PrE: previous pregnancy with preeclampsia, CVD: cardiovascular disease, HTN: hypertension

**Table S3:** Regression models for association between % blood pressure dipping and demographics

| **Variables** | **% SBP dip** | | **% DBP dip** | | **% MAP dip** | |
| --- | --- | --- | --- | --- | --- | --- |
|  | **Unadjusted**  **β**  **(95% CI)** | **Adjusted***  **β**  **(95% CI)** | **Unadjusted**  **β**  **(95% CI)** | **Adjusted***  **β**  **(95% CI)** | **Unadjusted**  **β**  **(95% CI)** | **Adjusted***  **β**  **(95% CI)** |
| **Age**  **BMI at enrolment**  **Weight gain**  **GA at BP assessment**  **Parity**  **Number BP meds**  **Gestational diabetes**  **ESS**  **PSQI**  **Previous PrE**  **Family history of CVD**  **Category of HTN** | 0.03(-0.28,0.34)  -0.04(-0.21,0.14)  0.06(-0.23,0.34)  0.05(-0.26,0.37)  0.08(-1.43,1.59)  -0.78(-3.03,1.47)  -0.56(-2.83,1.71)  -0.19(-0.61,0.22)  -0.12(-0.57,0.34)  -0.30(-3.67,3.07)  0.29(-3.49,4.08)  -0.28(-4.07,3.50) | 0.04(-0.29,0.37)  -0.03(-0.22,0.16)  0.06(-0.26,0.37)  0.04(-0.31,0.38  0.01(-1.62,1.64)  -0.81(-3.19,1.58)  -0.92(-3.62,1.79)  -0.14(-0.61,0.34)  -0.12(-0.62,0.38)  -0.22(-3.95,3.52)  0.59(-3.56,4.73)  -0.64(-5.24,3.97) | 0.00(-0.46,0.46)  -0.19(-0.44,0.07)  0.28(-0.09,0.64)  0.40(-0.05,0.85)  0.61(-1.63,2.86)  -0.83(-4.20,2.54)  -1.99(-5.34,1.36)  -0.46(-1.09,0.16)  -0.57(-1.17,0.03)  -0.14(-5.17,4.88)  -1.14(-6.78,4.50)  0.48(-5.16,6.13) | -0.02(-0.50,0.45)  -0.14(-0.42,0.13)  0.27(-0.13,0.68)  0.33(-0.16,0.83)  0.07(-2.27,2.41)  -0.62(-4.05,2.80)  -2.18(-6.03,1.67)  -0.38(-1.07,0.32)  -0.64(-1.32,0.05)  -0.89(-6.25,4.47)  -0.71(-6.68,5.25)  -0.41(-7.03,6.21) | -0.01(-0.39,0.38)  -0.13(-0.35,0.09)  0.14(-0.18,0.47)  0.23(-0.15,0.62)  0.24(-1.65,2.12)  -1.52(-4.31,1.27)  -0.89(-3.73,1.94)  -0.41(-0.92,0.09)  -0.35(-0.89,0.19)  -1.08(-5.27,3.12)  -0.01(-4.74,4.72)  0.60(-4.12,5.32) | -0.01(-0.41,0.40)  -0.11(-0.34,0.12)  0.15(-0.21,0.50)  0.18(-0.23,0.60)  -0.10(-2.08,1.88)  -1.42(-4.32,1.48)  -1.18(-4.46,2.10)  -0.32(-0.90,0.25)  -0.38(-0.96,0.21)  -1.32(-5.84,3.20)  0.50(-4.54,5.54)  -0.19(-5.79,5.41) |

*Adjusted with age, BMI at enrolment, parity, number of BP medications, Gestational age at BP assessment.

% Blood pressure dip = 100 x (daytime-nighttime blood pressure)/daytime blood pressure

SBP: systolic blood pressure, DBP: diastolic blood pressure, MAP: mean arterial pressure,

BMI: body mass index, Weight gain: gestational weight gain, GA: gestational age, BP: blood pressure, ESS: Epworth Sleepiness Score, PSQI: Pittsburgh sleep quality index, Previous PrE: previous pregnancy with preeclampsia, CVD: cardiovascular disease, HTN: hypertension

**Table S4:** Heart rate characteristics of participants stratified by blood pressure dipping status.

|  | **Systolic blood pressure** | | | **Diastolic blood pressure** | | | **Mean arterial pressure** | | |
| --- | --- | --- | --- | --- | --- | --- | --- | --- | --- |
|  | **Dippers**  **(n=11)** | **Nondippers**  **(n=40)** | **p-value** | **Dippers**  **(n=21)** | **Nondippers**  **(n=30)** | **p-value** | **Dippers**  **(n=18)** | **Nondippers**  **(n=33)** | **p-value** |
| 24h HR, bpm | 90.7±6.3 | 88.7±9.1 | 0.49 | 89.5±6.1 | 88.9 ±10.0 | 0.79 | 74.4± 11.9 | 75.0± 9.9 | 0.63 |
| 24h HR SD | 8.7±1.7 | 8.5±3.4 | 0.87 | 8.4±2.7 | 8.6±3.3 | 0.79 | 8.5±2.9 | 8.6±3.2 | 0.90 |
| 24h HR CoV, % | 9.6±1.9 | 9.6±3.8 | 0.97 | 9.5±3.3 | 9.8±3.7 | 0.76 | 9.5±3.5 | 9.7±3.5 | 0.84 |
| Daytime HR, bpm | 92.4±7.1 | 90.1±9.4 | 0.46 | 91.1±6.7 | 90.2±10.4 | 0.72 | 91.4±7.1 | 90.2±9.9 | 0.61 |
| Daytime HR SD | 8.7±1.8 | 8.3±3.4 | 0.71 | 8.3±2.8 | 8.4±3.3 | 0.96 | 8.4±3.0 | 8.3±3.2 | 0.90 |
| Daytime HR CoV, % | 9.4±2.1 | 9.2±3.6 | 0.84 | 9.2±3.4 | 9.3±3.4 | 0.94 | 9.3±3.6 | 9.2±3.2 | 0.92 |
| Nighttime HR, bpm | 86.5±5.3 | 85.3±9.3 | 0.59 | 85.8±5.4 | 85.4±10.3 | 0.88 | 86.2±5.3 | 85.2±10.0 | 0.64 |
| Nighttime HR SD | 6.2±2.1 | 6.7±4.3 | 0.75 | 5.8±2.7 | 7.2±4.6 | 0.22 | 5.7±2.7 | 7.0±4.4 | 0.26 |
| Nighttime HR CoV, % | 7.3±2.8 | 7.9±5.1 | 0.72 | 6.8±3.3 | 8.4±5.4 | 0.24 | 6.7±3.3 | 8.3±5.3 | 0.26 |

Values are expressed as mean ± standard deviation

p-value for dippers vs. nondippers groups

24h = 24 hours, HR= heart rate, SD= standard deviation, bpm= beats per minute

CoV= coefficient of variability was calculated from standard deviation of heart rate divided by mean of heart rate x 100%

**Table S5:** Correlations between coefficient of variability of heart rate and obstructive sleep apnea severity measures

|  | **24h HR CoV**  **r (p-value)** | **Daytime HR CoV**  **r (p-value)** | **Nighttime HR CoV**  **r (p-value)** |
| --- | --- | --- | --- |
| AHI  REM-AHI  4% ODI  Resp arousal index  Nadir SpO_2_  TST<90% | -0.204(0.15)  -0.260(0.08)  -0.104(0.47)  -0.296(0.05)  0.076(0.60)  -0.089(0.54) | -0.196(0.17)  -0.238(0.11)  -0.101(0.49)  -0.293(0.05)  0.095(0.51)  -0.090(0.53) | -0.061(0.67)  -0.143(0.34)  -0.036(0.80)  -0.074(0.62)  -0.056(0.70)  -0.002(0.99) |

r = Pearson’s correlation coefficient

CoV= coefficient of variability was calculated from standard deviation of heart rate divided by mean of heart rate x 100%

AHI: apnea-hypopnea index, REM-AHI: apnea-hypopnea index during REM sleep, 4%ODI: 4% oxygen desaturation index, Resp arousal index: respiratory arousal index, SpO_2_: oxygen saturation during sleep, TST < 90% = total sleep time with oxygen saturation < 90%

**Table S6:** Patient characteristics stratified by carotid femoral pulse wave velocity (cfPWV) status.

|  | **Total**  **(n=43)** | **High cfPWV**  **(n=22)** | **low cfPWV**  **(n=21)** |
| --- | --- | --- | --- |
| **Maternal Age, years** | 36.3±5.2 | 36.1±4.0 | 36.5±6.4 |
| **Pre-pregnancy BMI, kg/m^2^** | 33.3±7.6 | 35.3±7.0 | 30.9±7.7 |
| **BMI at enrolment, kg/m^2^** | 35.2±6.9 | 37.2±6.8 | 33.1±6.5 |
| **Gestational weight gain, kg** | 5.8±5.9 | 6.1±5.4 | 5.5±6.9 |
| **GA at arterial stiffness, weeks** | 28.0±4.8 | 28.6±5.5 | 27.4±4.0 |
| **GA at sleep study, weeks** | 25.1±5.1 | 25.9±5.5 | 24.4±4.2 |
| **ESS** | 9.7±3.3 | 10.1±3.4 | 9.3±3.3 |
| **PSQI** | 8.9±3.7 | 8.6±3.0 | 9.1±4.2 |
| **Parity**  Nulliparous  Multiparous | 14(32.6%)  29(67.4%) | 6(27.3%)  16(72.7%) | 8(38.1%)  13(61.9%) |
| **Category of hypertension**  Chronic hypertension  Gestational hypertension | 34(79.1%)  9 (20.9%) | 17(77.3%)  5(22.7%) | 17(81.0%)  4(19.0%) |
| **Anti-hypertensive medications:**  Hydralazine  Methyldopa  Labetalol  Nifedipine  Enalapril  **Other Medications:**  ASA  Insulin  Metformin | 1(2.3%)  9(20.9%)  29(67.4%)  22(51.2%)  1(2.3%)  36(83.7%)  5(11.6%)  3(7.0%) | 1(4.5%)  4(18.2%)  17(77.3%)  11(50.0%)  1(4.5%)  19(86.4%)  2(9.1%)  0 | 0  5(23.8%)  12(57.1%)  11(52.4%)  0  17(81.0%)  3(14.3%)  3(14.3%) |
| **Number of BP medications**  1  2  $\geq$3 | 28(65.1%)  12(27.9%)  3(7.0%) | 13(59.1%)  7(31.8%)  2(9.0%) | 15(71.4%)  5(23.8%)  1(4.8%) |
| **Diabetes status**  Gestational diabetes  Pre-existing diabetes | 7(16.3%)  3(7.0%) | 5(22.7%)  0 | 2(9.5%)  3(14.3%) |
| **Ethnicity**  Caucasian  African American  Other | 21(48.8%)  9(20.9%)  13(30.2%) | 11(50.0%)  6(27.3%)  5(22.7% | 10(47.6%)  3(14.3%)  8(38.1%) |
| **Previous pregnancy with PrE** | 13(30.2%) | 7(31.8%) | 6(28.6%) |
| **Family history of CVD** | 33(76.7%) | 19(86.4%) | 14(66.7%) |
| **Severity of OSA**  Mild  Moderate  Severe | 17(39.5%)  22(51.2%)  4(9.3%) | 9(40.9%)  11(50.0%)  2(9.1%) | 8(38.1%)  11(52.4%)  2(9.5%) |

Values are presented in means ± SD or numbers (%). BMI: body mass index, GA: gestational age, BP: blood pressure, ESS: Epworth Sleepiness Score, PSQI: Pittsburgh sleep quality index, PrE: preeclampsia, CVD: cardiovascular disease, OSA: obstructive sleep apnea **Table S7:** Regression models for association between carotid femoral pulse wave velocity and 24-hour blood pressure measures

|  | **Carotid femoral pulse wave velocity** | |
| --- | --- | --- |
|  | **Unadjusted β**  **(95% CI)** | **Adjusted β***  **(95% CI)** |
| **24-hour SBP**  **24-hour DBP**  **24-hour MAP**  **Daytime SBP**  **Daytime DBP**  **Daytime MAP**  **Nighttime SBP**  **Nighttime DBP**  **Nighttime MAP**  **% SBP dip**  **% DBP dip**  **% MAP dip** | **0.04(0.002,0.07)**  0.02(-0.02,0.06)  0.03(-0.01,0.07)  **0.04(0.01,0.07)**  0.02(-0.02,0.06)  0.04(-0.01,0.07)  0.03(0.00,0.06)  0.01(-0.03,0.05)  0.02(-0.02,0.05)  0.01(-0.07,0.08)  0.02(-0.04,0.07)  0.02(-0.04,0.08) | **0.05(0.02,0.08)**  **0.05(0.01,0.09)**  **0.06(0.02,0.09)**  **0.05(0.02,0.08)**  **0.05(0.01,0.09)**  **0.06(0.03,0.10)**  **0.04(0.01,0.07)**  **0.04(0.001,0.07)**  **0.04(0.01,0.07)**  -0.02(-0.09,0.04)  0.00(-0.04,0.05)  0.00(-0.05,0.06) |

*Adjusted with age, parity, gestational age at BP assessment and BMI at enrolment.

SBP: systolic blood pressure, DBP: diastolic blood pressure, MAP: mean arterial pressure,

% Blood pressure dip = 100 x (daytime-nighttime blood pressure)/daytime blood pressure

**Figure S1:** Linear regression models demonstrated association between carotid femoral pulse wave velocity and body mass index at enrolment


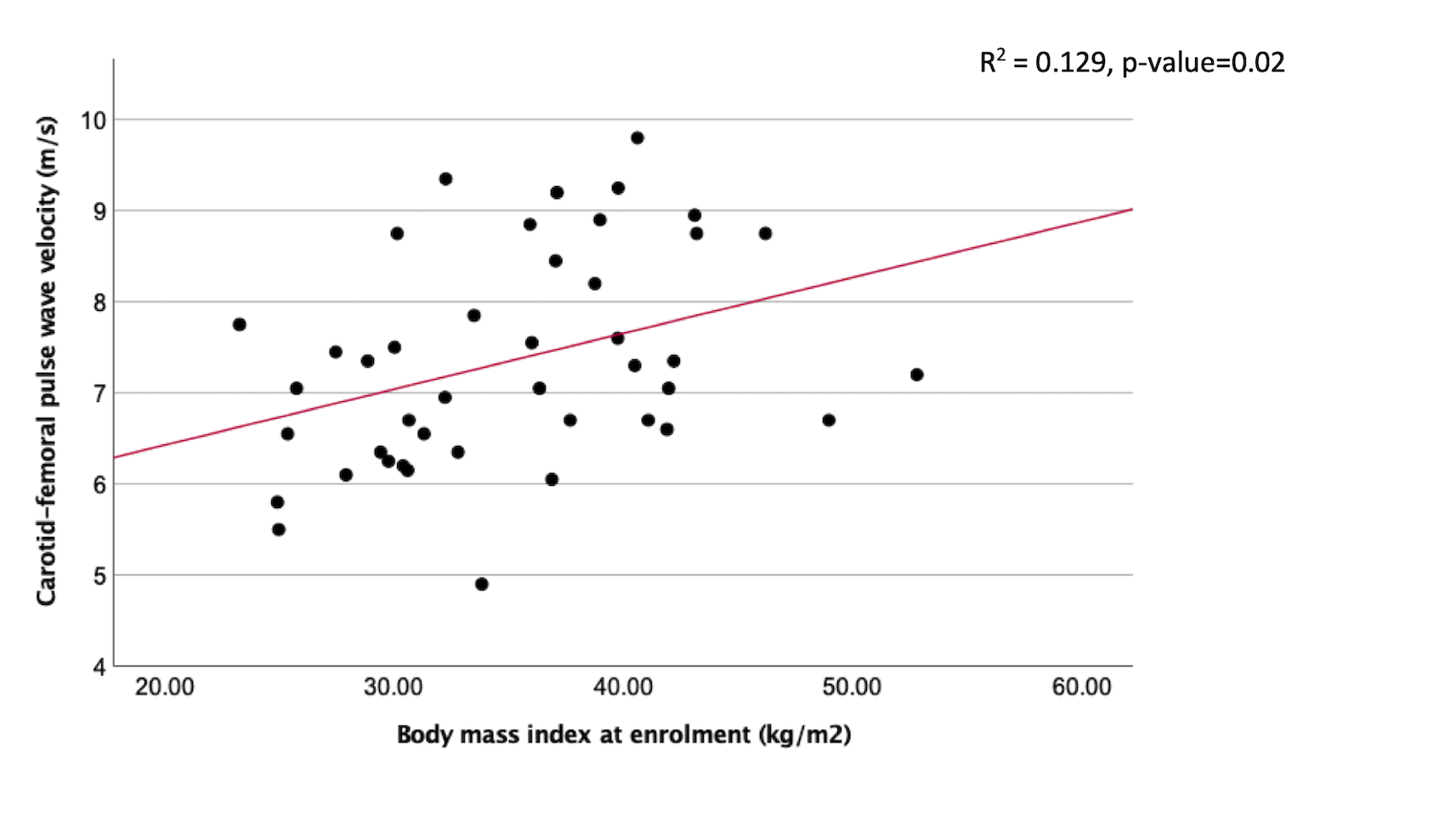

Supplement: Supplementary file 1 [file Data_Sheet_1.docx]
